# Andrographolide, A Plant‐Based Dietary Bioactive Nutritional Health Compound, Attenuates Hyperoxia‐Induced Acute Lung Injury by Inhibiting Ferroptosis Through the STAT3/P53 Signaling Pathway

**DOI:** 10.1002/fsn3.72513

**Published:** 2026-11-09

**Authors:** Qiuyuan Xia, Xinxin Liu, Song Qin, Banghai Feng, Kun Yu, Yingcong Ren

**Affiliations:** ^1^ Department of Critical Care Medicine Luzhou People's Hospital Luzhou Sichuan China; ^2^ Department of Critical Care Medicine Affiliated Hospital of Zunyi Medical University Zunyi Guizhou China; ^3^ Department of Critical Care Medicine Zunyi Hospital of Traditional Chinese Medicine Zunyi Guizhou China; ^4^ Department of Geriatrics The First Affiliated Hospital of Chengdu Medical College Chengdu Sichuan China

**Keywords:** andrographolide, dietary bioactive compounds, ferroptosis, functional food, hyperoxia‐induced acute lung injury, nutritional and health products, P53, STAT3

## Abstract

Andrographolide (AG) is a plant‐derived bioactive diterpenoid compound with potential applications in nutritional science and clinical nutrition. This study investigated whether AG attenuates hyperoxia‐induced acute lung injury (HALI) by inhibiting ferroptosis via the signal transducer and activator of transcription 3 (STAT3)/tumor protein P53 (P53) signaling pathway. Male C57BL/6 mice were used to establish a hyperoxia‐induced lung injury model, and the protective effects of AG were compared with those of the ferroptosis inhibitor ferrostatin‐1 (Fer‐1). Lung histopathological changes, the lung wet‐to‐dry (W/D) weight ratio, reactive oxygen species (ROS), ferrous iron (Fe^2+^), glutathione (GSH), inflammatory cytokines, and glutathione peroxidase 4 (GPX4) expression were assessed. Network pharmacology and functional enrichment analyses were performed to identify potential targets of AG, followed by validation in MLE‐12 lung epithelial cells and mouse models. Cell viability was assessed using the CCK‐8 assay, and the expression of phosphorylated STAT3 (P‐STAT3), total STAT3, P53, and GPX4 was examined by western blotting. Co‐immunoprecipitation (Co‐IP) was used to investigate the interaction between STAT3 and P53. Hyperoxia induced marked lung injury, inflammatory cell infiltration, increased pulmonary edema, and alterations in ferroptosis‐related indicators. Both AG and Fer‐1 attenuated lung injury and reduced ferroptosis‐associated changes. Network pharmacology analysis identified 79 potential targets associated with HALI, with STAT3 emerging as a core candidate target. In MLE‐12 cells, AG attenuated ferroptosis‐associated changes, whereas the STAT3 phosphorylation activator Colivelin TFA attenuated these protective effects. Co‐IP confirmed an interaction between STAT3 and P53, and Colivelin TFA also weakened the protective effects of AG in the mouse model. These findings suggest that AG may alleviate HALI by inhibiting ferroptosis through modulation of the STAT3/P53 signaling pathway, providing experimental evidence for its potential as a plant‐derived bioactive compound for further investigation in hyperoxia‐induced lung injury.

## Graphical abstract

Andrographolide (AG) attenuates hyperoxia‐induced acute lung injury (HALI) by modulating the STAT3/P53 signaling pathway and ferroptosis‐related responses. AG treatment reduces STAT3 phosphorylation and P53 expression, increases GPX4 expression, and alleviates oxidative stress, iron accumulation, and inflammatory responses, thereby protecting alveolar epithelial cells and ameliorating lung injury. Colivelin TFA partially reverses these protective effects, suggesting that modulation of STAT3/P53 signaling may contribute to the protective effects of AG against HALI. The graphical abstract was prepared using Figdraw.

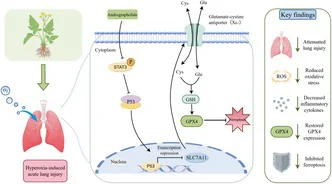

## 1 Introduction

Hyperoxia‐induced acute lung injury (HALI) is a severe complication of oxygen therapy in critically ill patients and can progress to acute respiratory distress syndrome (ARDS) and bronchopulmonary dysplasia (BPD). The incidence of HALI in intensive care units (ICUs) is approximately 12%–18%, with a mortality rate of 35%–40% (Lilien et al. 2023; Bellani et al. 2016). Currently, there are no effective therapeutic strategies for HALI, and clinical management remains limited to prevention and supportive care only. Injury to alveolar epithelial cells and pulmonary vascular endothelial cells constitutes the major pathological basis for HALI (Qin, Zhuang, et al. 2023; Qin, Liu, et al. 2023). Therefore, investigating the protective mechanisms targeting these cells is of great significance for clinical treatment of HALI.

In addition to being used as a pharmacological agent, andrographolide represents a broader class of plant‐based dietary bioactive compounds, whose regulation of metabolic and cell death pathways is increasingly receiving attention from the nutritional science community (Dai et al. 2019). The role of phytochemicals from dietary sources, including diterpenes, polyphenols, and other functional food ingredients, in regulating iron homeostasis, lipid peroxidation, and redox metabolism, has been increasingly recognized. These processes are the core mechanisms of ferroptosis and link daily dietary exposure to disease risk in critically ill patient populations (Zheng et al. 2021). Therefore, elucidating the effects of dietary bioactive compounds on these metabolic pathways not only deepens our understanding of the pharmacological mechanisms of acute lung injury but also contributes to a broader understanding of the interactions between nutrition metabolism and disease.

Ferroptosis is a non‐apoptotic form of regulated cell death characterized by iron‐dependent lipid peroxidation, which can be triggered by iron overload, dysregulated amino acid metabolism, and lipid peroxide accumulation (Dixon and Olzmann 2024). Recent studies have shown that ferroptosis plays a crucial role in the development and progression of acute lung injuries. Treatment with the ferroptosis inhibitor ferrostatin‐1 (Fer‐1) suppresses ferroptosis in human bronchial epithelial cells and alleviates lipopolysaccharide (LPS)‐induced acute lung injury (Liu et al. 2020). Recent evidence also indicates that the inhibition of alveolar epithelial cell ferroptosis ameliorates lung injury in smoke exposure‐induced chronic obstructive pulmonary disease (COPD) (Fan et al. 2023). In sepsis‐associated acute lung injury, neutrophil extracellular traps (NETs) promote ferroptosis in alveolar epithelial cells by regulating hypoxia‐inducible factor‐1 methylation (Zhang et al. 2023). In HALI, the regulation of IL‐17A expression can inhibit ferroptosis in alveolar epithelial cells, thereby attenuating lung injury (Guo et al. 2022). Our previous study also demonstrated that apoptosis and ferroptosis are the major forms of alveolar epithelial cell death in HALI (Li, Wang, et al. 2024). These findings suggest that ferroptosis is an important mechanism underlying HALI. Accordingly, identifying drugs that can inhibit ferroptosis is of great importance in the treatment of HALI.

Traditional Chinese medicine provides abundant resources for the development of effective therapeutic agents. Increasing evidence has shown that the extraction and identification of active compounds from traditional Chinese medicine using modern technologies is an important research focus (Shang, Shen, Stub, Zhu, et al. 2022; Zhu et al. 2022). The therapeutic effects of traditional Chinese medicine in acute lung injury‐related diseases, including coronavirus disease 2019, have been widely recognized (Ye et al. 2021). Our previous study also found that traditional Chinese medicine exerts protective effects against HALI (Li, Wang, et al. 2024). Andrographolide (AG) is a major bioactive diterpenoid isolated from *Andrographis paniculata* , an edible medicinal plant widely consumed as herbal tea, dietary supplement, and functional food ingredient in many Asian countries. Owing to its potent antioxidant, anti‐inflammatory, and immunomodulatory activities, AG has attracted increasing attention as a dietary bioactive compound with potential health‐promoting effects (Zeng et al. 2022; Dai et al. 2024). Pharmacokinetic studies have further demonstrated that orally administered AG can be absorbed into the systemic circulation, although its oral bioavailability is relatively limited because of extensive first‐pass metabolism, supporting its potential as an orally administered dietary bioactive ingredient (Zeng et al. 2022). Previous studies have demonstrated that AG alleviates sepsis‐induced acute lung injury by promoting macrophage autophagy (Qin et al. 2024). In addition, AG has been reported to target TLR4 and regulate the Keap1/Nrf2 pathway, thereby inhibiting ferroptosis and inflammatory responses in LPS‐induced acute lung injury (Li, Huang, et al. 2024). However, AG has multiple potential targets, and its precise mechanisms remain unclear, particularly in cases of HALI. Therefore, elucidating the protective effects of AG in HALI and its molecular mechanisms may provide new insights into the treatment of HALI.

From a clinical and public health nutrition perspective, andrographolide‐containing preparations derived from *Andrographis paniculata* are widely used in capsules, tablets, and other dietary or herbal products. Clinical studies have generally reported good short‐term tolerability of oral Andrographis preparations, including those providing relatively high oral doses of andrographolide, although mild and reversible adverse events may occur (Shang, Shen, Stub, et al. 2022). Due to the deterioration of nutritional status and increased oxidative stress in critically ill patients receiving long‐term oxygen therapy, bioactive compounds derived from food, such as AG, represent a class of easily implementable and potentially transformative clinical nutritional interventions. Therefore, in regions where *Andrographis paniculata* ‐based products have been incorporated into routine dietary practices, evaluating dietary supplements containing AG as an adjunct to standard supportive treatment may be of special significance. Simultaneously, this highlights the broader differences in the accessibility and standardization of such dietary supplements in different medical environments.

Therefore, we hypothesized that AG exerts protective effects against HALI by inhibiting ferroptosis in alveolar epithelial cells. In this study, we established a mouse model of HALI induced by high‐concentration oxygen exposure (FiO_2_ ≥ 90%) and an in vitro model of hydrogen peroxide‐stimulated alveolar epithelial cells to clarify the protective effects of AG against HALI and explore the molecular mechanisms by which AG regulates alveolar epithelial cell ferroptosis. The dose of andrographolide used in mice (20 mg/kg) fell within the pharmacologically effective range reported in previous preclinical studies. According to body surface area‐based conversion recommended for interspecies dose extrapolation, this dose corresponds to approximately 1.6 mg/kg/day in humans (~100 mg/day for a 60‐kg adult), which is comparable to the range of andrographolide exposure achievable from standardized *Andrographis paniculata* dietary supplements (Zeng et al. 2022; Reagan‐Shaw et al. 2008). These findings may provide novel experimental evidence for the development of therapeutic strategies for HALI.

## 2 Materials and Methods

### 2.1 Chemicals and Reagents

The chemical reagents used in this study included andrographolide (AG; Cat. No. A7330, Solarbio) and the ferroptosis inhibitor ferrostatin‐1 (Fer‐1; Cat. No. HY‐100579 (MCE) and the STAT3 phosphorylation activator Colivelin TFA (Cat. No. HY‐P1061A, MCE). The primary antibodies used were anti‐GPX4 (Cat. No. 52455; CST) and anti‐STAT3 (cat. No. 9139, CST), anti‐phospho‐STAT3 (P‐STAT3; Cat. No. 9145, CST), anti‐P53 (cat. No. 2524, CST), and anti‐β‐actin (cataloged as No. 4970, CST). Goat anti‐rabbit and anti‐mouse secondary antibodies were purchased from CST (Cat. Nos. 7074 and 7076). ELISA kits for TNF‐α (Cat. No. E‐EL‐M3063), and IL‐1β (Cat. No. E‐EL‐M0037) and IL‐6 (Cat. No. E‐EL‐M0044) were obtained from Elab Sciences. ROS assay kits (Cat. No. EEA019) and Fe^2+^ assay kits (Cat. No. EEA008) and GSH assay kits (Cat. No. EEA020) were purchased from ThermoFisher Scientific. The CCK‐8 assay kit was purchased from Abcam (Cambridge, UK). No. ab228554). Australian premium fetal bovine serum (FBS; Cat. No. 10099141C) and DMEM/F12 (Cat. No. 11320033) were purchased from Gibco.

### 2.2 Experimental Animals and Establishment of the Animal Model

This study was approved by the Animal Ethics Committee of the Affiliated Hospital of Zunyi Medical University (Approval No. zyfy‐an‐202400443). Healthy male C57BL/6 mice aged 6–8 weeks and weighing 20 ± 2 g were purchased from the Experimental Animal Center of Zunyi Medical University (License No. SYXK [Qian] 2021‐0004). All mice were housed in a specific pathogen‐free (SPF) facility at the Biological Research Center of Zunyi Medical University. The room temperature was maintained at 18°C–22°C, with a relative humidity of 50%–60% and a 12‐h light/dark cycle. The mice had free access to food and water ad libitum. Our previous study demonstrated that exposure of mice to hyperoxia (FiO_2_ ≥ 90%) for 48 h successfully established a HALI animal model (Liu et al. 2019).

According to previously reported protocols, mice were pretreated before HALI model establishment by intraperitoneal injection of AG (20 mg/kg, every 12 h for 3 days), Fer‐1 (1 mg/kg, once daily for 14 days), or Colivelin TFA (1 mg/kg, once daily for 14 days) (Qin et al. 2024; Ling et al. 2023; Yao et al. 2024). After establishing the model, the mice were euthanized for further analysis.

### 2.3 Cell Culture and Establishment of the Cell Model

Mouse lung epithelial cells (MLE‐12; Cat. No. CRL‐2110 (ATCC)) cells were maintained in a medium supplemented with 10% FBS. Cells were passaged until the fifth generation and used in subsequent experiments. Our previous study confirmed that treatment of MLE‐12 cells with 0.5 mM hydrogen peroxide for 8 h successfully established an in vitro HALI cell model (Liu, Dai, et al. 2025).

According to previously reported protocols, when MLE‐12 cells reached 60%–70% confluence, they were pretreated with AG (1 μg/mL) for 4 h or Colivelin TFA (50 μg/mL) for 24 h before establishing the model (Qin et al. 2024; Qian et al. 2025).

### 2.4 Histopathological Analysis of Lung Tissue

After euthanasia, the lower lobe of the left lung was excised. Excess moisture was removed using absorbent paper, and the tissue was cut into small pieces of approximately 1 mm. The samples were fixed in 4% paraformaldehyde, embedded, sectioned, and stained with hematoxylin and eosin (H&E). Pathological changes were observed under a light microscope and scored according to previously described criteria (Kulkarni et al. 2022).

### 2.5 Enzyme‐Linked Immunosorbent Assay

The concentrations of TNF‐α, IL‐1β, IL‐6, GSH, and Fe^2+^ in the cell culture supernatants and lung tissue homogenates were measured using ELISA kits according to the manufacturer's instructions. Briefly, samples and reagents were equilibrated to room temperature, and 100 μL of the standards or diluted samples were added to each well. After incubation at room temperature for 90 min, a biotinylated antibody working solution was added and incubated for 1 h. After three washes, an enzyme‐conjugate working solution was added and incubated at room temperature for 30 min. Substrate solution was then added, and the plates were incubated in the dark for 15 min. Finally, a stop solution was added to terminate the reaction, and the optical density was measured using a microplate reader.

### 2.6 Measurement of Reactive Oxygen Species

ROS levels in lung tissue homogenates or cells were measured using the 2′, 7′‐dichlorodihydrofluorescein diacetate (DCFH‐DA) fluorescent probe method according to the instructions provided with the ROS assay kit. Lung tissues were homogenized, and protein concentrations were determined using the BCA assay for normalization. The cells were seeded in 96‐well plates before detection. Fluorescence intensity was measured using a microplate reader at excitation and emission wavelengths of 502/525 nm.

### 2.7 Measurement of Lung Wet/Dry Weight Ratio

After euthanasia, the upper lobe of the left lung was excised. Excess surface moisture was removed with absorbent paper, and the tissue was weighed to obtain the wet weight. The tissue was then dried at 60°C for 48 h and weighed again to obtain the dry weight. The lung wet/dry weight (W/D) ratio was then calculated.

### 2.8 Cell Viability Assay

MLE‐12 cells were seeded into 96‐well plates at a density of 1 × 10^5^ cells/mL with 100 μL per well and cultured for 48 h. After replacing the medium with fresh medium, 90 μL of medium and 10 μL of the CCK‐8 working solution were added to each well. The cells were incubated for 3 h, and the optical density was measured using a microplate reader. Cell viability was calculated according to the manufacturer's instructions.

### 2.9 Western Blotting

Lung tissues and MLE‐12 cells were lysed using lysis buffer. Total proteins were separated by 8% or 12% SDS‐PAGE and transferred onto PVDF membranes. The membranes were blocked with 2% skim milk for 2 h and then incubated overnight at 4°C with primary antibodies against GPX4, SLC7A11, STAT3, P‐STAT3, P53, and β‐actin. After incubation with secondary antibodies at room temperature for 2 h, the protein bands were visualized using ECL reagent and exposed to a gel imaging system. Images were analyzed using the ImageJ software. The relative expression levels of the target proteins were determined based on the gray values of the bands, with β‐actin as the internal control.

### 2.10 Co‐Immunoprecipitation

The cells were lysed to release the total proteins. Specific antibodies were added to the lysates to bind the target proteins, followed by incubation with protein A/G agarose beads to capture the antibody–protein complex. After washing to remove unbound proteins, the bound proteins were eluted and analyzed by SDS‐PAGE and Western blotting.

### 2.11 Network Pharmacology Analysis

The natural analogs of andrographolide include neoandrographolide, 14‐deoxy‐11, 12‐didehydroandrographolide, deoxyandrographolide, isoandrographolide, 14‐deoxy‐11‐oxo‐andrographolide, and 14‐deoxyandrographolide.

The predicted targets of AG were obtained from the TCMSP (https://tcmsp.91medicine.cn), PubChem (https://pubchem.ncbi.nlm.nih.gov), SwissTargetPrediction (http://www.swisstargetprediction.ch), and TCMIP v2.0 online databases (http://www.tcmip.cn). HALI‐related targets were retrieved from GeneCards (https://www.genecards.org), OMIM (https://omim.org), TTD (https://ttd.idrblab.cn), and DrugBank (http://www.drugbank.ca). The overlapping targets between the AG‐related‐ and HALI‐related targets were identified and used to construct a new target gene set. Gene Ontology (GO) and Kyoto Encyclopedia of Genes and Genomes (KEGG) enrichment analyses were performed using the DAVID database and an online bioinformatics platform (http://www.bioinformatics.com.cn/), with the significance threshold set at *p* < 0.05. Protein–protein interaction (PPI) network analysis of the target genes was performed using the STRING database. The species were restricted to humans ( *Homo sapiens* ), and a confidence score of > 0.7 was required to ensure data quality.

### 2.12 Molecular Docking

To further investigate the interaction and potential binding mechanism between STAT3 and AG, molecular docking was performed to predict their binding affinities and modes. Three‐dimensional structural models of AG and STAT3 were obtained from the PubChem database (https://pubchem.ncbi.nlm.nih.gov/). These models were imported into AutoDock software (AutoDockTools‐1.5.7) for docking analysis to evaluate the binding activity. The docking results were subsequently visualized and analyzed using PyMOL software (PyMOL 2.3.2).

### 2.13 Statistical Analysis

Statistical analyses were performed using SPSS 29.0, and data visualization was conducted using GraphPad Prism 9.0. The experimental results are presented as mean ± standard error of the mean (SEM). Each experiment was performed at least three times. Comparisons among multiple groups were performed using one‐way analysis of variance (ANOVA), followed by Dunnett's post hoc test for multiple comparisons. Statistical significance was set at *p* < 0.05.

## 3 Results

### 3.1 AG Exerts Anti‐Inflammatory Effects, Inhibits Ferroptosis, and Alleviates Lung Injury in HALI Mice

Our previous studies demonstrated that exposure of mice to hyperoxia (FiO_2_ ≥ 90%) for 48 h successfully established a HALI animal model and that Fer‐1 inhibits ferroptosis in HALI (Li, Wang, et al. 2024; Liu et al. 2019). To investigate the effects of AG on lung injury and ferroptosis during HALI, we used the classical ferroptosis inhibitor Fer‐1 as a positive control and evaluated lung histopathological injury, W/D ratio, inflammatory cytokine levels, oxidative stress, Fe^2+^ concentration, and GPX4 expression in the lung tissues of HALI mice.

Histopathological examination and W/D analysis showed that after 48 h of hyperoxia exposure, the mice exhibited alveolar wall thickening, increased alveolar exudation, extensive neutrophil infiltration, and pulmonary edema. Fer‐1 treatment significantly ameliorated lung histopathological injury. Similar to Fer‐1, AG markedly attenuated lung injury in HALI mice (Figure 1A–C). Consistent results were observed in the assessment of inflammatory cytokines and oxidative stress. Lung tissues from hyperoxia‐exposed mice showed increased levels of IL‐1β, IL‐6, TNF‐α, ROS, and Fe^2+^, accompanied by decreased GSH levels. AG treatment significantly reduced IL‐1β, IL‐6, TNF‐α, ROS, and Fe^2+^ levels, while increasing GSH levels in the lung tissues of HALI mice, and these effects were comparable to those observed after Fer‐1 treatment (Figure 1D,E).

**FIGURE 1:**
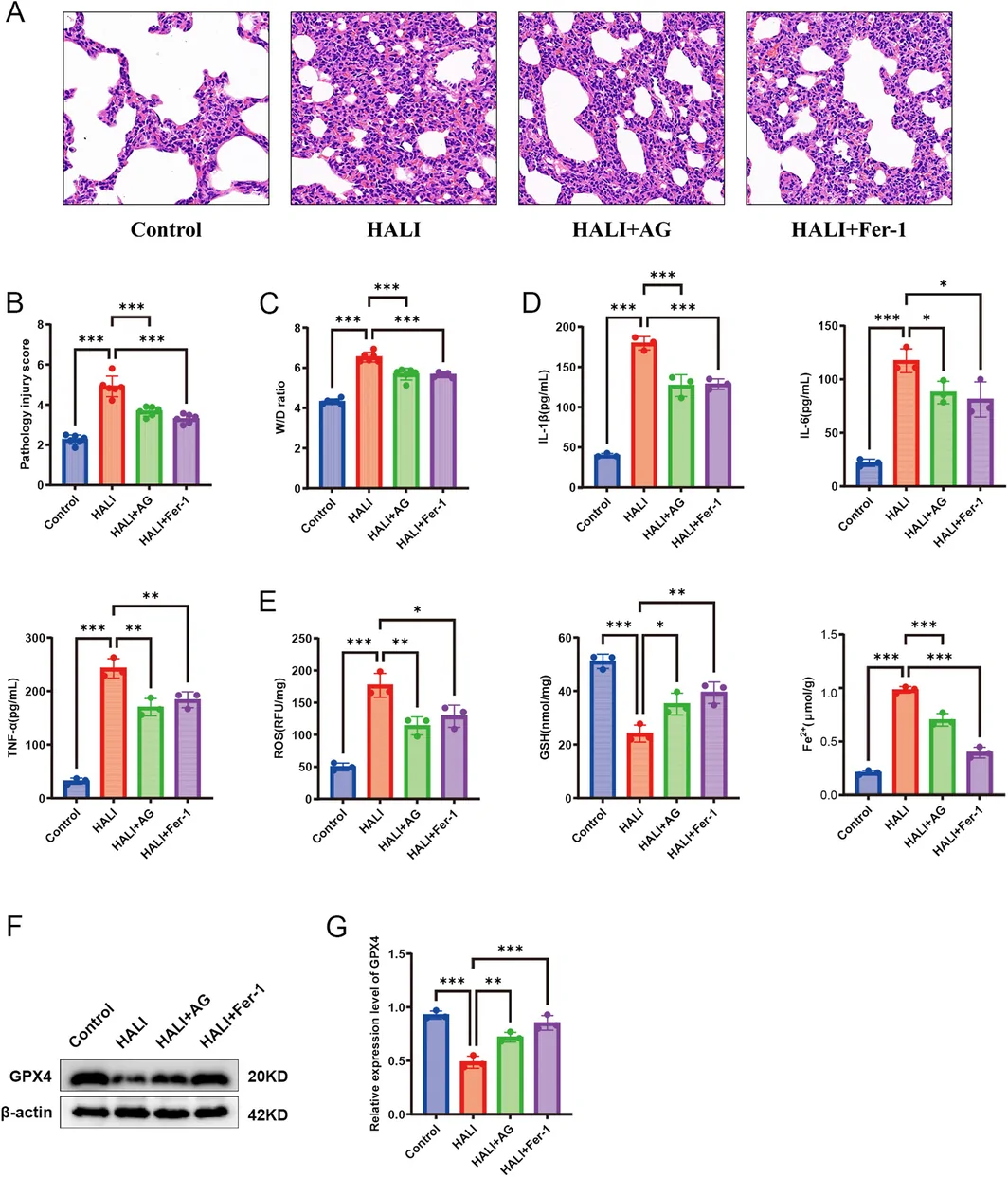
AG exerts anti‐inflammatory effects, inhibits ferroptosis, and alleviates lung injury in HALI mice. (A, B) Representative H&E staining images of lung tissues (40×) and pathological lung injury scores. (C) Lung wet/dry weight ratio (W/D ratio). (D) IL‐1β, IL‐6 and TNF‐α levels in lung tissues. (E) ROS levels, GSH content and Fe^2+^ concentration in the lung tissues. (F, G) GPX4 expression levels in the lung tissue. Data are presented as mean ± SEM (*n* = 3). The significance threshold was set at *p* < 0.05. *p < 0.05, **p < 0.01, ***p < 0.001.

GPX4 is a key antioxidant enzyme in the ferroptosis pathway that primarily functions to suppress ferroptosis in cells (Huang et al. 2025). At the protein level, GPX4 expression was decreased in the lung tissues of HALI mice, whereas both AG and Fer‐1 treatments upregulated GPX4 expression (Figure 1F,G). Together, these results indicate that, similar to Fer‐1, AG suppresses inflammatory responses, oxidative stress, and ferroptosis, thereby alleviating lung injury in HALI.

### 3.2 Network Pharmacology Analysis of AG and HALI

To further elucidate the potential mechanisms by which AG protects against lung injury in HALI, we performed a network pharmacology analysis using TCMSP, PubChem, SwissTargetPrediction, TCMIP v2.0, GeneCards, OMIM, TTD, and DrugBank. Seven active components of AG were identified. In addition, 305 targets associated with these AG‐related active components and 1017 HALI‐related targets were identified. By intersecting these two target sets, 79 overlapping targets were identified as potential targets of AG in the treatment of HALI (Figure 2A,D).

**FIGURE 2:**
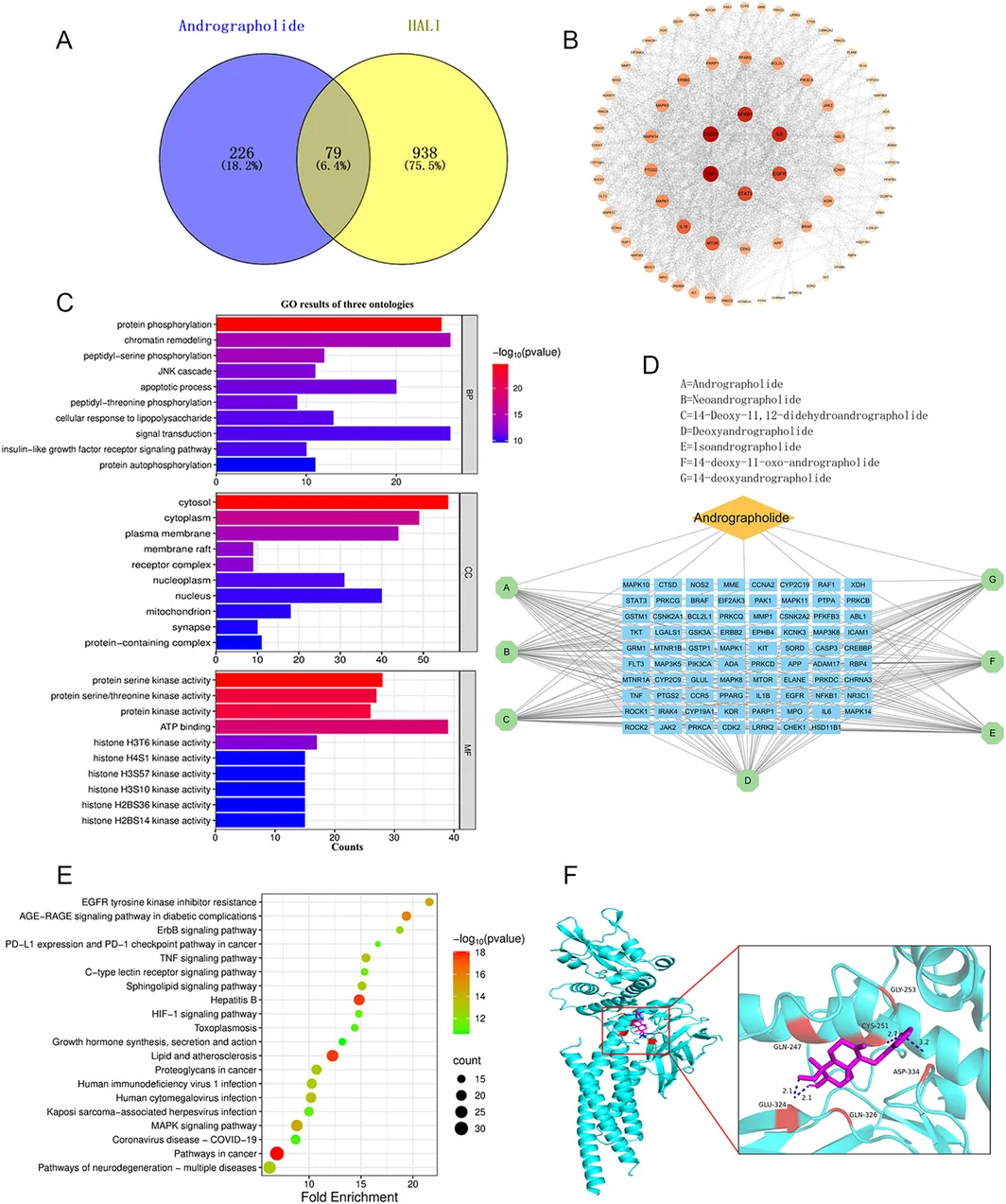
Network pharmacology analysis of AG and HALI. (A) Venn diagram showing overlapping targets between AG and HALI‐related diseases. (B) PPI network constructed using STRING database. (C) GO functional enrichment analysis of the overlapping targets of AG and HALI. (D) Network of AG‐related active components and their targets. (E) KEGG pathway enrichment analysis of overlapping targets between AG and HALI. (F) Molecular docking model of AG and STAT3.

A protein–protein interaction (PPI) network was constructed to identify key targets. The top‐ranked core targets included TNF, CASP3, IL6, NFKB1, EGFR, and STAT3 (Figure 2B). GO and KEGG pathway enrichment analyses were performed (Figure 2C,E). Biological process (BP) analysis showed that these genes were mainly associated with cellular signal transduction, oxidative stress, post‐translational modification, particularly phosphorylation, and programmed cell death. In terms of cellular components (CCs), these genes were primarily associated with the cytoplasmic matrix, plasma membrane, nucleus and mitochondria. Molecular function (MF) analysis indicated that the targets were mainly involved in kinase activity, particularly protein phosphorylation activity.

KEGG pathway enrichment analysis showed that these genes were involved in pathways related to cancer, lipid metabolism, atherosclerosis and oxidative stress. The top 20 significantly enriched pathways were selected based on their enrichment significance. Among them, the lipid and atherosclerosis, AGE‐RAGE, MAPK, and TNF signaling pathways were the key pathways involved in oxidative stress and lipid metabolism. Based on the PPI and MF results, we focused on the phosphorylation modification of STAT3, which was enriched in the aforementioned pathways and may mediate oxidative stress and lipid metabolism.

Molecular docking was performed to predict the interaction strength and potential binding modes of AG and STAT3. The results showed that AG could bind to GLU‐324, CYS‐251, GLN‐326, GLY‐253, GLN‐247, and ASP‐334 residues in STAT3 with a binding energy of −7.2 kcal/mol (Figure 2F).

### 3.3 AG Exerts Anti‐Inflammatory and Antioxidant Effects in a Hydrogen Peroxide‐Induced Oxidative Injury Model of MLE‐12 Cells

Our previous study demonstrated that treatment of MLE‐12 cells with 0.5 mM hydrogen peroxide for 8 h successfully established an in vitro HALI cell model (Qian et al. 2025). To evaluate whether AG exerts its effects through STAT3, we used the STAT3‐specific activator Colivelin TFA as an experimental control in the HALI cell models. Hydrogen peroxide stimulation markedly increased the levels of IL‐1β, IL‐6, TNF‐α, ROS, and Fe^2+^, while reducing the GSH levels in MLE‐12 cells. Treatment with AG significantly attenuated these alterations by decreasing the production of IL‐1β, IL‐6, TNF‐α, ROS, and Fe^2+^ and restoring GSH levels. Conversely, the addition of Colivelin TFA abolished the protective effects of AG on oxidative stress‐ and inflammation‐related changes (Figure 3A,B). Consistently, AG markedly alleviated hydrogen peroxide‐induced cellular injury, whereas Colivelin TFA significantly weakened the cytoprotective effect of AG (Figure 3C).

**FIGURE 3:**
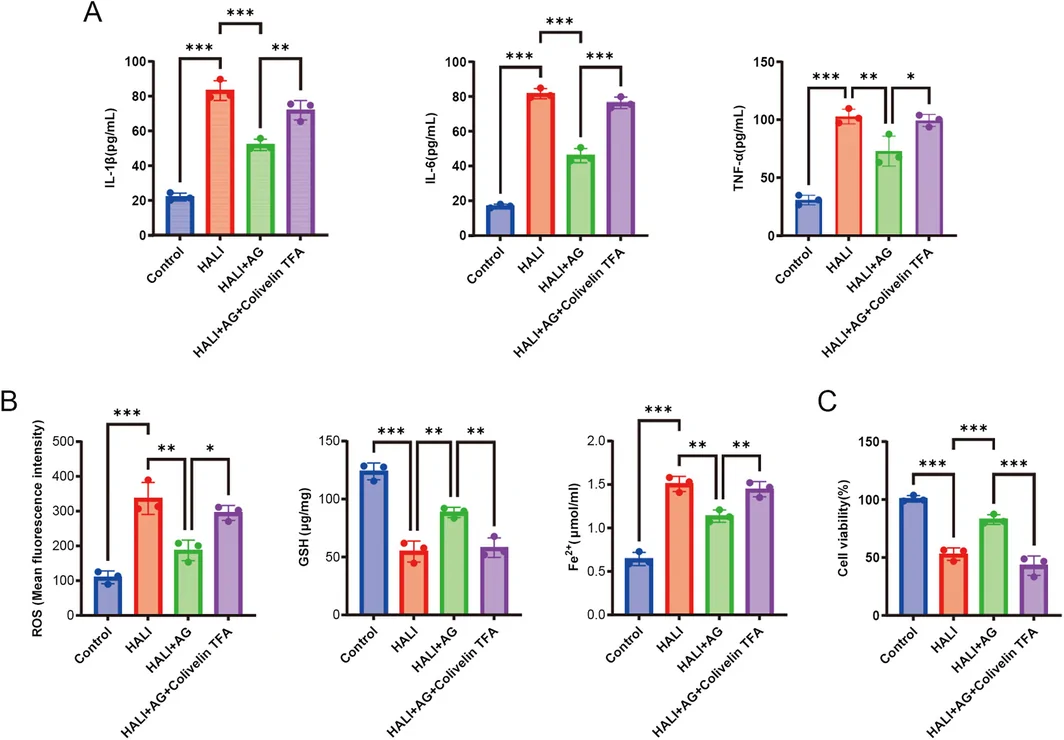
AG exerts anti‐inflammatory and antioxidant effects in MLE‐12 cells. (A) IL‐1β, IL‐6, and TNF‐α levels in MLE‐12 cells. (B) ROS levels, GSH, and Fe^2+^ concentrations in MLE‐12 cells. (C) Cell viability was assessed using the CCK‐8 assay. Data are presented as mean ± SEM (*n* = 3). The significance threshold was set at *p* < 0.05. *p < 0.05; **p < 0.01; ***p < 0.001.

### 3.4 AG Inhibits Ferroptosis in MLE‐12 Cells Through the STAT3/P53 Signaling Pathway

To further determine the expression of STAT3 and its downstream targets in HALI and their changes after AG intervention, we examined the levels of related proteins using western blotting and Co‐immunoprecipitation (Co‐IP). Western blotting showed that P‐STAT3 expression was increased after MLE‐12 cell injury (Figure 4A,B), which was consistent with our previous findings (Qin, Liu, et al. 2023; Mei et al. 2022). Injured MLE‐12 cells also exhibited upregulated P53 expression and downregulated GPX4 expression levels. AG treatment decreased the levels of P‐STAT3 and P53, while increasing the expression of GPX4. However Colivelin TFA blocked these effects of AG (Figure 4A,B).

**FIGURE 4:**
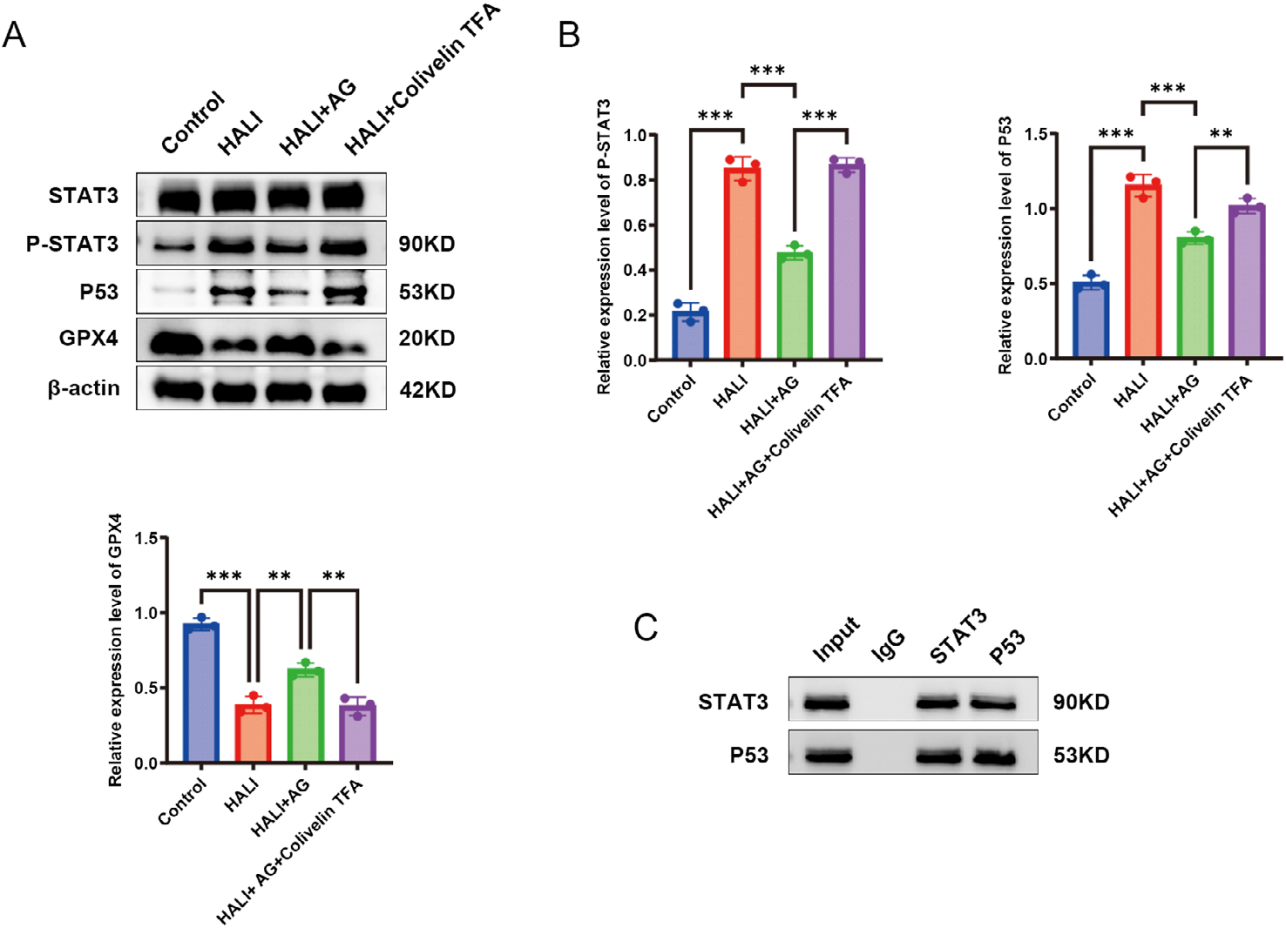
AG inhibits ferroptosis in MLE‐12 cells via the STAT3/P53 signaling pathway. (A, B) Representative Western blot images and quantitative analysis of P‐STAT3, P53 and GPX4 expression in MLE‐12 cells. (C) Co‐IP analysis of the interaction between STAT3 and P53. Data are presented as the mean ± SEM (*n* = 3). The significance threshold was set at 0.05. **p < 0.01, ***p < 0.001.

To further clarify the relationship between STAT3 and P53, Co‐IP analysis was performed, which confirmed an endogenous interaction between STAT3 and P53 (Figure 4C).

### 3.5 The Lung‐Protective Effect of AG in HALI Mice Is Associated With the STAT3/P53 Signaling Pathway

To investigate whether the lung‐protective effects of AG in inhibiting oxidative stress and ferroptosis during HALI are associated with STAT3, we continued to use Colivelin TFA as an experimental control. The results showed that Colivelin TFA blocked the protective effects of AG, as evidenced by alveolar wall thickening, increased alveolar exudation, inflammatory cell infiltration, and aggravated lung injury (Figure 5A–C).

**FIGURE 5:**
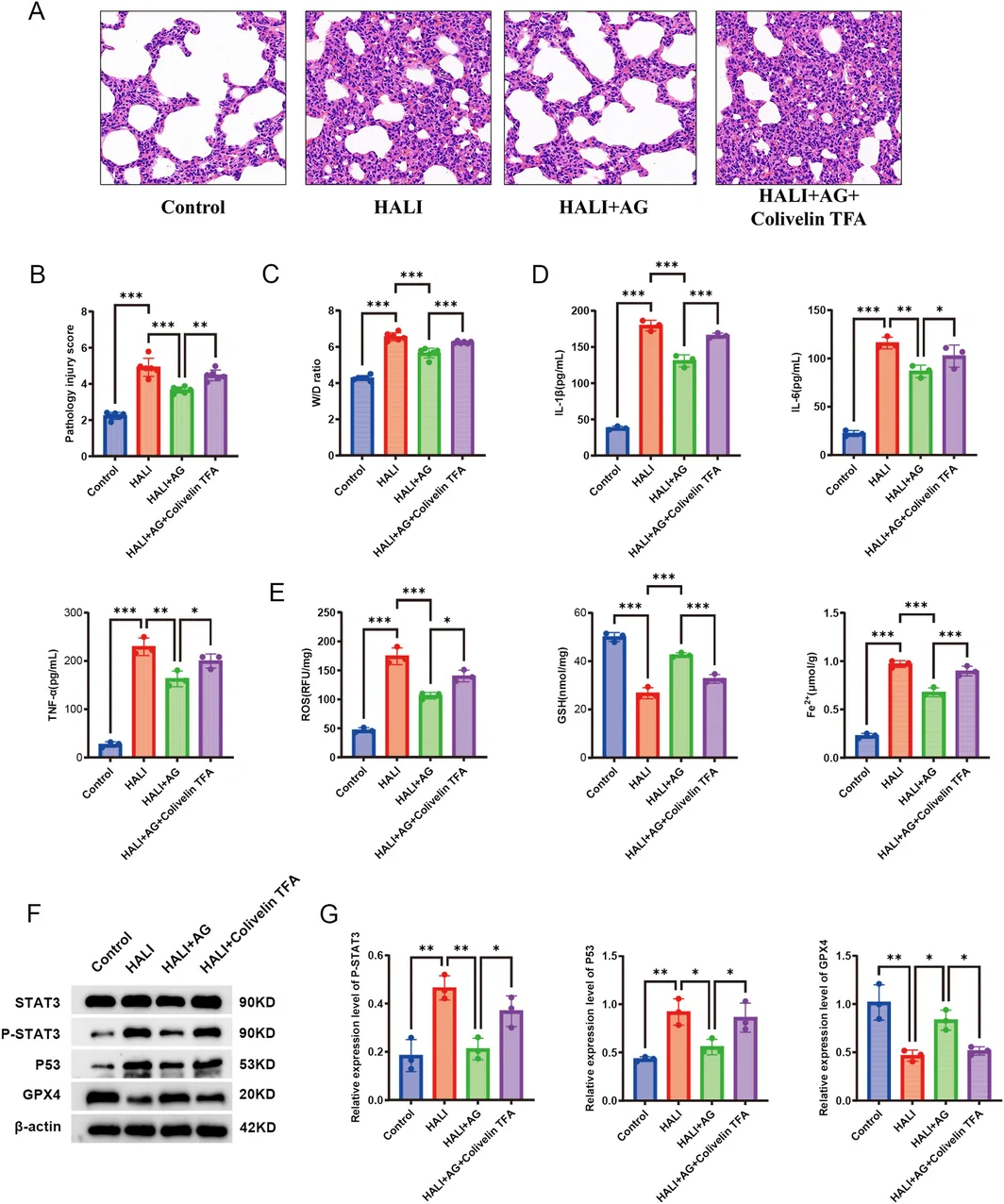
The lung‐protective effect of AG in HALI mice was associated with the STAT3/P53 signaling pathway. (A, B) Representative H&E staining images of lung tissues (40×) and pathological lung injury scores. (C) Lung wet/dry weight (W/D) ratio. (D) Levels of IL‐1β, IL‐6, and TNF‐α in lung tissues. (E) ROS levels, GSH content, and Fe^2+^ concentration in lung tissues. (F, G) Representative Western blot images and quantitative analysis of P‐STAT3, P53 and GPX4 expression in lung tissue. Data are presented as the mean ± SEM (*n* = 3). The significance threshold was set at 0.05. *p < 0.05; **p < 0.01; ***p < 0.001.

Consistent findings were observed in the assessment of inflammatory cytokines and oxidative stress. Lung tissues from Colivelin TFA‐treated mice showed increased levels of IL‐1β, IL‐6, TNF‐α, ROS, and Fe^2+^, accompanied by decreased GSH levels (Figure 5D,E). Similarly, at the protein level, Colivelin TFA reversed the effects of AG, as evidenced by decreased GPX4 expression and increased P‐STAT3 and P53 levels in mouse lung tissues (Figure 5F,G). These findings suggest that the lung‐protective effects of AG may be associated with the regulation of STAT3/P53 signaling and ferroptosis‐related responses in the lung.

## 4 Discussion

HALI is a severe complication of prolonged high‐concentration oxygen therapy in critically ill patients. It can progress to ARDS or BPD and is associated with high morbidity and mortality; however, specific therapeutic strategies remain lacking (Lilien et al. 2023; Bellani et al. 2016; Zhu et al. 2024). The pathogenic mechanisms of HALI are complex and have not yet been fully elucidated. HALI is characterized by excessive pulmonary inflammatory responses, immune cell infiltration, pro‐inflammatory cytokine release, and extensive injury to endothelial and epithelial cells (Qin, Zhuang, et al. 2023; Liu, Jia, et al. 2025). Therefore, exploring the pathological mechanisms of HALI and identifying effective intervention targets are of considerable clinical significance.

These physiological processes have special significance for nutritional metabolism, as cellular iron processing, glutathione‐dependent antioxidant capacity, and polyunsaturated fatty acid peroxidation—the core biochemical drivers of iron death—are directly influenced by dietary intake and nutritional status. Dietary antioxidants and trace element cofactors can regulate the GSH/GPX4 axis discussed in this study, thereby positioning iron death as a mechanistic node of the interaction between nutritional factors and acute organ injury. Therefore, our research findings expand the current understanding of how bioactive compounds from food sources act on metabolic cell death pathways regulated by nutritional factors, providing relevant evidence for the broader field of nutritional regulation of metabolic pathways and disease risks.

Alveolar epithelial cells serve as the first physical barrier against pathogen invasion and participate in immune surveillance by expressing various pattern recognition receptors (PRRs). These receptors recognize microbial components and activate the innate immune system (Kumar 2020). The importance of these protective mechanisms has prompted efforts to harness the defensive capacity of alveolar epithelial cells to prevent and treat HALI (Kallinos et al. 2025; Yi et al. 2025). Therefore, in the present study, we used the alveolar epithelial cell line, MLE‐12, to establish an in vitro model. Given the current lack of effective therapeutic options, screening natural medicinal plant extracts as novel strategies for the prevention and management of HALI is urgently required.

Traditional Chinese medicine has become a research focus because of its unique natural advantages (Shang, Shen, Stub, Zhu, et al. 2022; Zhu et al. 2022; Ye et al. 2021), and our previous studies have confirmed the lung‐protective effects of several traditional Chinese medicines (Li, Wang, et al. 2024). AG is a major dietary bioactive compound extracted from the traditional Chinese medicinal herb *Andrographis paniculata* , exhibiting multiple pharmacological activities, including anti‐inflammatory, antioxidant, anti‐apoptotic, and immunomodulatory effects (Zeng et al. 2022; Dai et al. 2024). Recent studies have shown that AG exerts clear lung‐protective effects in multiple acute lung injury (ALI) models (Qin et al. 2024; Li, Huang, et al. 2024; Cheng et al. 2025). These studies mainly focused on the anti‐inflammatory and antioxidant effects of AG. However, the full therapeutic potential of AG in HALI and its precise underlying mechanisms remain unclear.

In the present study, we demonstrated that AG markedly alleviated HALI. AG restored key physiological parameters, including the lung W/D ratio and histopathological changes, reflecting ALI severity. In addition, both in vivo and in vitro experiments showed that AG pre‐treatment significantly suppressed the expression of pro‐inflammatory mediators, including IL‐6, IL‐1β, and TNF‐α, in HALI models. To systematically reveal the mechanism by which AG treats HALI, we performed network pharmacology analysis to identify the pathways shared by AG and HALI. GO and KEGG enrichment analyses indicated that STAT3 may be a core target through which AG exerts its effects on HALI. STAT3 is an important signal transducer and activator of transcription that plays key roles in cellular signal transduction, gene expression regulation, and various physiological and pathological processes (Chen et al. 2025). Previous studies have demonstrated that STAT3 is an important regulator of ferroptosis, and its phosphorylation activates ferroptosis (Xie et al. 2025).

Ferroptosis is an emerging form of regulated cell death characterized by the interplay between oxidative stress, iron metabolism, and lipid peroxidation (Dixon and Olzmann 2024). Our previous study identified ferroptosis as a key factor in the pathogenesis of HALI (Li, Wang, et al. 2024). In the present study, hyperoxia exposure significantly increased Fe^2+^ and ROS levels in mouse lung tissues while reducing GSH content and GPX4 expression, suggesting the activation of ferroptosis. AG intervention reversed these changes, and its effects were comparable to those of the classical ferroptosis inhibitor Fer‐1, indicating that AG exerts strong anti‐ferroptotic effects. This finding is consistent with a previous report showing that AG alleviates LPS‐induced acute lung injury by regulating the Keap1/Nrf2 pathway to inhibit ferroptosis (Li, Huang, et al. 2024), suggesting that AG may exert a shared anti‐ferroptotic effect in acute lung injury induced by different stimuli.

This study further confirmed through cellular experiments that AG significantly inhibited hydrogen peroxide‐induced STAT3 phosphorylation and downregulated the expression of the downstream protein P53. Co‐IP analysis verified the interaction between STAT3 and P53, suggesting that they may form a functional complex involved in the regulation of ferroptosis. As a classical tumor suppressor protein, P53 inhibits the transcription of solute carrier family 7 member 11 (SLC7A11), leading to ROS accumulation, impaired GSH synthesis, reduced antioxidant capacity, and ultimately ferroptosis (Jiang et al. 2015). Sustained STAT3 phosphorylation can bind to the S100A6 promoter and upregulate P53 expression, thereby suppressing SLC7A11 expression and promoting ferroptosis (Yang et al. 2025). The results of the present study are consistent with these previous studies. After AG intervention, the levels of P‐STAT3 and P53 decreased, whereas the expression of GPX4 increased, indicating that cellular ferroptosis was inhibited. Treatment with the STAT3 phosphorylation activator Colivelin TFA reversed the protective effects of AG, further confirming the critical role of the STAT3/P53 pathway in the inhibition of ferroptosis.

Notably, crosstalk between STAT3 and P53 has been reported in fields such as oncology; however, its underlying mechanisms are complex and remain incompletely understood (Pham et al. 2020). However, few studies have investigated the interaction between STAT3 and P53 in hyperoxia‐induced lung injury and their regulation of ferroptosis. To our knowledge, this study is the first to demonstrate that AG inhibits STAT3 phosphorylation in a HALI model, thereby affecting P53 signaling and ultimately upregulating the ferroptosis‐related protein GPX4. These findings provide new experimental evidence for understanding the role of the STAT3/P53 axis in lung injury (Figure 6). Network pharmacology and molecular docking analyses predicted favorable binding activity between AG and STAT3, with a binding energy of −7.2 kcal/mol, providing theoretical support for STAT3 as a potential direct target of AG treatment.

**FIGURE 6:**
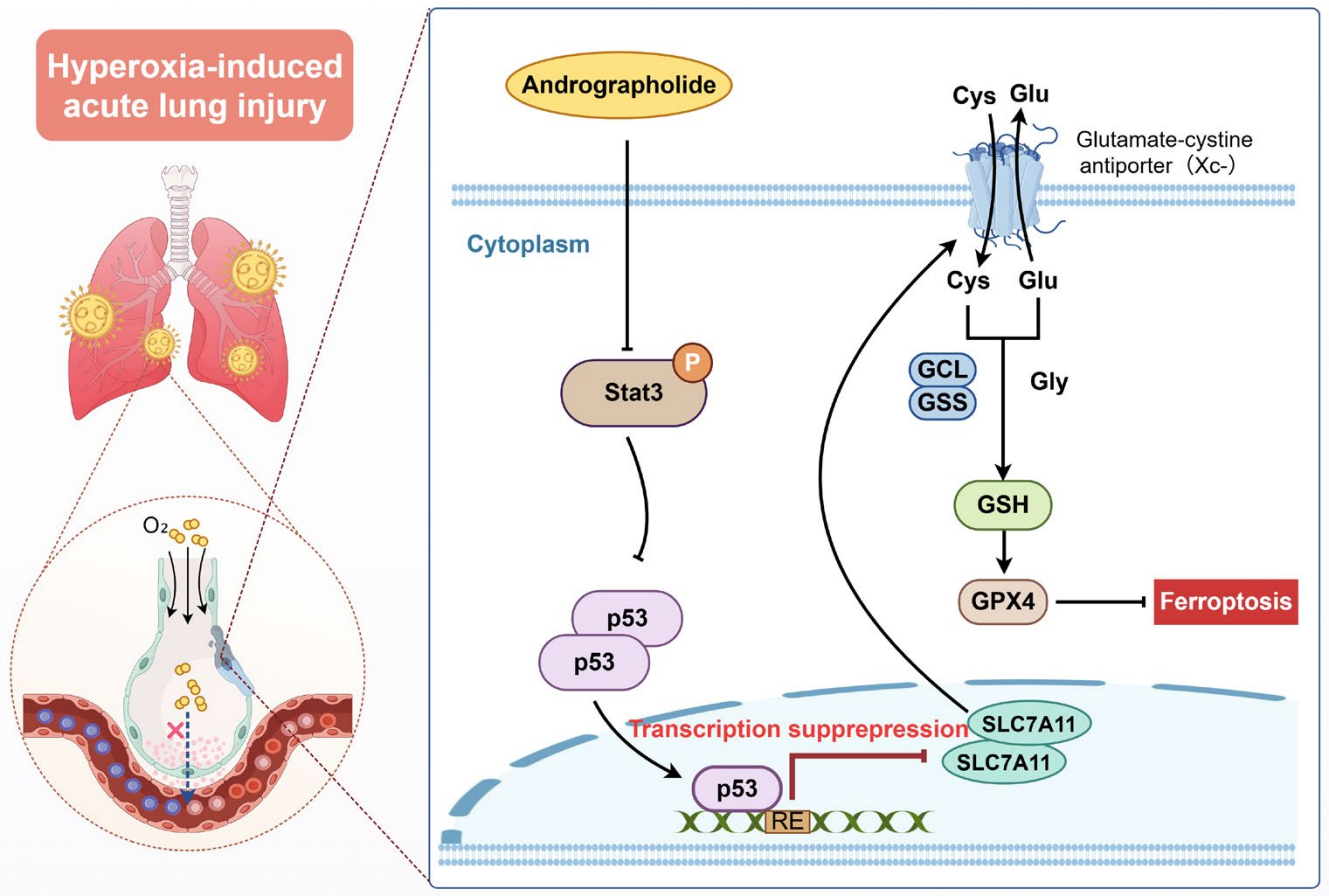
Schematic illustration of the mechanism by which AG ameliorates HALI by inhibiting ferroptosis via the STAT3/P53 signaling pathway.

The innovation of this study lies in the systematic elucidation of the molecular mechanism by which AG inhibits ferroptosis in alveolar epithelial cells through the STAT3/P53 signaling pathway, thereby attenuating HALI. These findings provide important experimental evidence to support the potential clinical application of AG. However, this study had several limitations. First, although network pharmacology identified STAT3 as a core target, whether AG directly binds to and inhibits STAT3 requires further validation using techniques such as surface plasmon resonance and mass spectrometry. Second, although Co‐IP analysis confirmed the interaction between STAT3 and P53, the structural basis of this interaction and the underlying transcriptional regulatory mechanisms involved in ferroptosis remain unclear. Moreover, the present study did not investigate how the STAT3–P53 interaction directly regulates ferroptosis‐related genes. Future studies are needed to clarify the detailed molecular mechanisms underlying this process. Finally, hydrogen peroxide induces oxidative stress and has been widely used as an in vitro surrogate model of hyperoxia. However, it cannot fully reproduce continuous oxygen exposure in vivo, which should be addressed in future studies using direct hyperoxic cell‐culture systems.

## 5 Conclusion

In summary, our findings suggest that AG may alleviate hyperoxia‐induced ferroptosis in alveolar epithelial cells by inhibiting STAT3/P53 signaling and upregulating GPX4 expression, thereby exerting protective effects against HALI. These findings provide experimental evidence supporting further investigation of AG as a potential protective compound for HALI.

## Author Contributions

**Qiuyuan Xia:** writing – original draft, conceptualization, methodology. **Xinxin Liu:** writing – original draft, conceptualization, methodology. **Song Qin:** writing – review and editing. **Banghai Feng:** data curation. **Kun Yu:** data curation. **Yingcong Ren:** writing – review and editing.

## Funding

This work was supported by grants from the National Natural Science Foundation of China (No. 82460373), Guizhou Provincial Department of Science and Technology (Nos. MS〔2026〕988, ZK‐2024‐299, ZK‐2024‐320), the Chengdu Medical College Start‐up Research Fund (No. CYFY‐GQ83), Science and Technology Fund of the Guizhou Provincial Health Commission (Nos. gzwkj2026‐179, gzwkj2024‐091), and Zunyi Science and Technology Bureau (No. HZ (2025) 312).

## Ethics Statement

All animal experiments were approved by the Animal Ethics Committee of the Affiliated Hospital of Zunyi Medical University, China (Approval No. zyfy‐an‐202400443). This study did not involve human participants or human data; therefore, informed consent was not applicable.

## Conflicts of Interest

The authors declare no conflicts of interest.

## Data Availability

The data that support the findings of this study are available from the corresponding author upon reasonable request.
